# Metastatic Nodular Melanoma with Angiosarcomatous Transdifferentiation—A Case Report and Review of the Literature

**DOI:** 10.3390/diagnostics14131323

**Published:** 2024-06-21

**Authors:** Adrian Vasile Dumitru, Dana Antonia Țăpoi, Mariana Costache, Ana Maria Ciongariu, Andreea Iuliana Ionescu, Horia Dan Liscu, Catalin Alius, Mircea Tampa, Andrei Marin, Andreea Roxana Furtunescu

**Affiliations:** 1 Department of Pathology, Carol Davila University of Medicine and Pharmacy, 020021 Bucharest, Romania; vasile.dumitru@umfcd.ro (A.V.D.); mariana.costache@umfcd.ro (M.C.); ana-maria.ciongariu@drd.umfcd.ro (A.M.C.); 2 Department of Pathology, University Emergency Hospital, 050098 Bucharest, Romania; 3 Department of Oncological Radiotherapy and Medical Imaging, Carol Davila University of Medicine and Pharmacy, 020021 Bucharest, Romania; andreea-iuliana.miron@drd.umfcd.ro (A.I.I.); horia-dan.liscu@drd.umfcd.ro (H.D.L.); 4 Department of Medical Oncology, Colțea Clinical Hospital, 030167 Bucharest, Romania; 5 Department of Radiotherapy, Colțea Clinical Hospital, 030167 Bucharest, Romania; 6 Faculty of Medicine, Carol Davila University of Medicine and Pharmacy Bucharest, 020021 Bucharest, Romania; catalin.alius@umfcd.ro; 7 Fourth Department of General Surgery, Emergency University Hospital Bucharest, 050098 Bucharest, Romania; 8 Department of Dermatology, Carol Davila University of Medicine and Pharmacy, 020021 Bucharest, Romania; mircea.tampa@umfcd.ro (M.T.); andreea-roxana.furtunescu@drd.umfcd.ro (A.R.F.); 9 Department of Dermatology, “Victor Babes” Clinical Hospital for Infectious Diseases, 030303 Bucharest, Romania; 10 Department of Plastic Surgery, Carol Davila University of Medicine and Pharmacy, 020021 Bucharest, Romania; andrei.marin@umfcd.ro

**Keywords:** cutaneous melanoma, angiosarcomatous, differentiation

## Abstract

Diagnosing cutaneous melanomas relies mainly on histopathological analysis, which, in selected cases, can be aided by immunohistochemical evaluation of conventional melanocytic markers. Nevertheless, these malignancies, particularly in metastatic settings, may display divergent differentiation with unusual histological and immunohistochemical features. In this context, we present the case of a 65-year-old male diagnosed with typical superficial spreading melanoma who developed recurrence and metastatic lesions featuring angiosarcomatous differentiation. The diagnosis of the initial tumour and the subsequently dedifferentiated lesions was confirmed by ample immunohistochemical analysis, which included several melanocytic markers, as well as mesenchymal and vascular markers. The recurrent tumour and lymph nodes metastases were completely negative for Melan-A and PRAME, and focally positive for SOX10. Additionally, they also displayed diffuse, intense positivity for CD10 and WT1 and focal positivity for CD99, ERB, and CD31. Thus, the diagnosis of primary cutaneous melanoma with recurrent and metastatic divergent angiosarcomatous differentiation was established. This occurrence is particularly rare and can pose important diagnostic challenges. Therefore, in addition to presenting this highly unusual case, we also performed a comprehensive review of the literature on divergent differentiation in melanomas.

## 1. Introduction

Cutaneous melanoma is the most aggressive form of skin cancer, in which survival is highly dependent on early and correct diagnosis [1]. Nevertheless, diagnosing primary and metastatic cutaneous melanomas is not always straightforward, as these tumours may display extraordinarily heterogenous histopathological and immunohistochemical features, including divergent differentiation, resembling various other neoplasms [2].

This is especially true of nodular melanoma (NM), which often lacks the clinical features of classic melanoma such as asymmetry, irregular borders, multiple colours, and dimensions greater than 0.6 cm. On dermatoscopy, atypical pigment network, regression structures, and irregular streaks are usually absent [3,4]. The diagnosis is further complicated by the fact that 20–30% of NMs are hypo- or amelanotic [3,5]. NM usually arises in previously normal skin, often in male patients over 50 years of age who do not have a personal or family history of skin cancer or other risk factors for melanoma development, such as freckles or numerous melanocytic naevi [5]. Moreover, NM is a fast-growing tumour with a high mitotic rate and the diagnosis is often made at advanced stages when the Breslow thickness of the tumour is already more than 2 mm [6,7,8,9]. Usually, the tumour is detected by the patient, and so far, the contribution of screening campaigns in the early detection of NM seems limited. Therefore, NM has a particularly poor prognosis and is associated with a disproportionately high mortality rate when compared with other melanoma subtypes [7]. 

Dermatoscopy can help raise the suspicion of NM and provide an earlier diagnosis of this aggressive tumour. NMs with a Breslow thickness of less than 2 mm are most often brown in colour and exhibit irregular brown dots or globules, irregular blue structureless areas, irregular eccentric black blotches, shiny white streaks, and dotted vessels. In more advanced NMs, asymmetry, blue colour, ulceration, and serpentine and corkscrew vessels become more common. There are three main features that can help in the diagnosis of thin NM: dotted vessels, shiny white streaks, and irregular blue structureless areas [3].

Some algorithms have been developed to aid in the diagnosis of NM. They can be used together with the ABCDE criteria in order to increase the specificity and sensitivity of NM diagnosis. The EFG rule refers to lesions that are elevated, firm, and growing [10], while the 3 Cs criteria evaluate colour, contour, and change [11]. The blue-black rule raises suspicion of melanoma in lesions that exhibit blue and black pigmentation in more than 10% of their surface [12]. 

The differential diagnosis of NM includes basal cell carcinoma; squamous cell carcinoma; Merkel cell carcinoma; melanocytic naevi, especially blue naevi and Spitz tumours; pyogenic granuloma; and atypical fibroxanthoma [6,13].

From a histopathological point of view, primary dedifferentiated cutaneous melanomas can be defined as biphasic tumours lacking conventional morphological and immunohistochemical characteristics while displaying non-melanocytic features [14]. Divergent differentiation, however, is more frequently noted in metastatic settings [15,16,17,18]. These tumours can present with widely variable morphologies and immune profiles and can be misdiagnosed as undifferentiated pleomorphic sarcoma, leiomyosarcoma, rhabdomyosarcoma, Ewing sarcoma, malignant peripheral nerve sheet tumour, poorly differentiated carcinoma, or, exceptionally rarely, angiosarcoma [14,16,19].

This phenomenon may represent a form of cancer plasticity aiding invasiveness and resistance to treatment [20,21]. Due to the presumed increased aggressiveness of dedifferentiated metastatic melanoma, establishing the correct diagnosis is tremendously important, but also a tedious process requiring extensive sampling, comprehensive immunohistochemical analysis, and even molecular tests for detecting genetic mutations typically associated with melanomas [14,22].

Given the rarity of dedifferentiated angiosarcomatous metastatic melanomas and their inherent diagnostic and management difficulties, we present the case of a superficial spreading melanoma with divergent angiosarcomatous differentiation in both locally recurrent and metastatic tumours. Furthermore, we performed an extensive review of previous published cases.

## 2. Materials and Methods

The tissue samples used for histopathological and immunohistochemical analyses were obtained after surgical removal of the primary cutaneous tumour and the recurrent cutaneous lesion, and lymphadenopathy. The tissue samples were fixed in 10% buffered formalin, paraffin-embedded, sectioned, and stained with Hematoxylin–Eosin (H&E) according to conventional histology protocols.

The sections used for immunohistochemistry were deparaffinised using toluene and alcohol, washed in phosphate saline buffer, incubated with normal serum, and later incubated with primary antibodies overnight. The secondary antibodies used were HMB45 (Biocare, mouse monoclonal, clone HMB45), PRAME (Biocare, rabbit monoclonal, clone EPR20330), SOX10 (Biocare, mouse monoclonal, clone BC34), Ki67 (Biocare, mouse monoclonal, clone MIB-1), desmin (Biocare, mouse monoclonal, clone D33), WT1 (Zeta, mouse monoclonal, clone 6F-H2), CD10 (Biocare, mouse monoclonal, clone 56C6), CD99 (Biocare, rabbit monoclonal, clone EP8), ERG (Biocare, mouse monoclonal, clone 9FY), CD31 (Biocare, mouse monoclonal, clone JC/70A), and BRAF V600E (Biocare, mouse monoclonal, clone VE1). The sections were developed using 3,3′-diaminobenzidine hydrochloride/hydrogen peroxide as a chromogen and counterstained with Meyer’s Haematoxylin.

Finally, we provided an extensive review of the literature by including complete-length English papers published until 2024 in PubMed-indexed journals discussing dedifferentiated melanomas, focusing on angionsarcomatous differentiation. All types of articles were included: reviews, original studies, and case reports. The research keywords were undifferentiated melanoma, dedifferentiated melanoma, transdifferentiated melanoma, and angiosarcomatous melanoma. 

## 3. Case Presentation

We present the case of a 65-year-old male who presented to our hospital in August 2021 with a pigmentary nodule on the upper posterior thorax. The tumour reportedly arose on previously normal skin about a year before, had been growing ever since, and had recently started to bleed. Clinical examination revealed an asymmetric pigmentary nodule of 1.2 × 0.9 cm, with well-demarcated borders and an uneven tumour surface. No on-transit or satellite metastases were seen, and palpation of the lymph nodes did not reveal any masses. A dermatoscopic examination revealed a blue colour, shiny white structures, and ulceration. The patient was otherwise well and did not have a personal or family history of melanoma or non-melanoma skin cancer. It was decided to excise the lesion, given its history of rapid growth and the clinical features.

**Figure 1:**
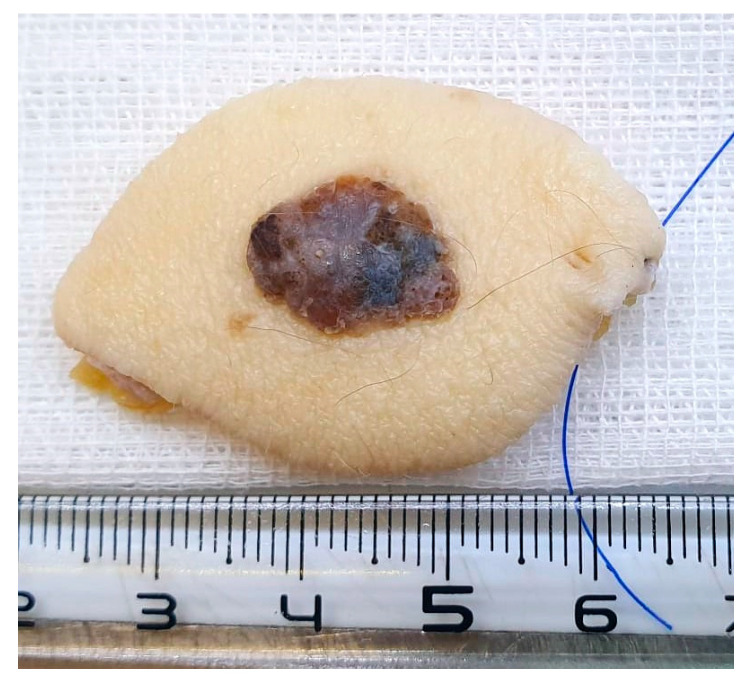
Post-excision photograph of a hyperpigmentary nodular melanoma, showing asymmetry, uneven surface, and ulceration.

On gross examination of the resection specimen, we noted the presence of a pigmented, nodular tumour with surface ulceration. Based on these findings, the clinical suspicion of a cutaneous melanoma was raised (Figure 1).

**Figure 2:**
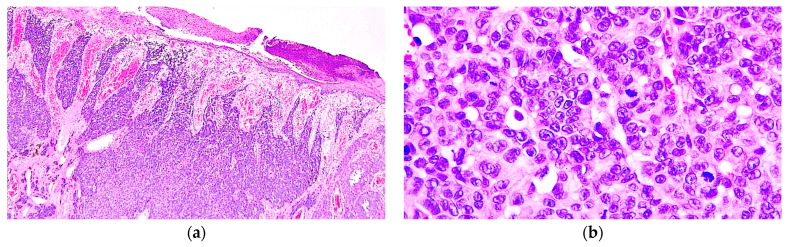
(**a**) Nodular melanoma composed of a solid growth pattern and epidermal ulceration (H&E, 40×). (**b**) Nests of epithelioid melanocytes with round nuclei displaying conspicuous nucleoli and frequent mitotic figures (H&E, 400×).

Microscopically, the tumour was composed of nests and solid areas of atypical epithelioid cells with focal intracytoplasmic melanin, enlarged nuclei with conspicuous nucleoli, and frequent mitotic figures (5/mm^2^). The tumour displayed a pagetoid growth pattern, ulcerating the epidermis, and was deeply invasive into the reticular dermis. The Breslow depth of invasion was 3.51 mm. No lympho-vascular invasion, perineural invasion, microsatellites, or necrotic areas were noted. Consequently, the diagnosis of pT3b nodular melanoma was established (Figure 2).

**Figure 3:**
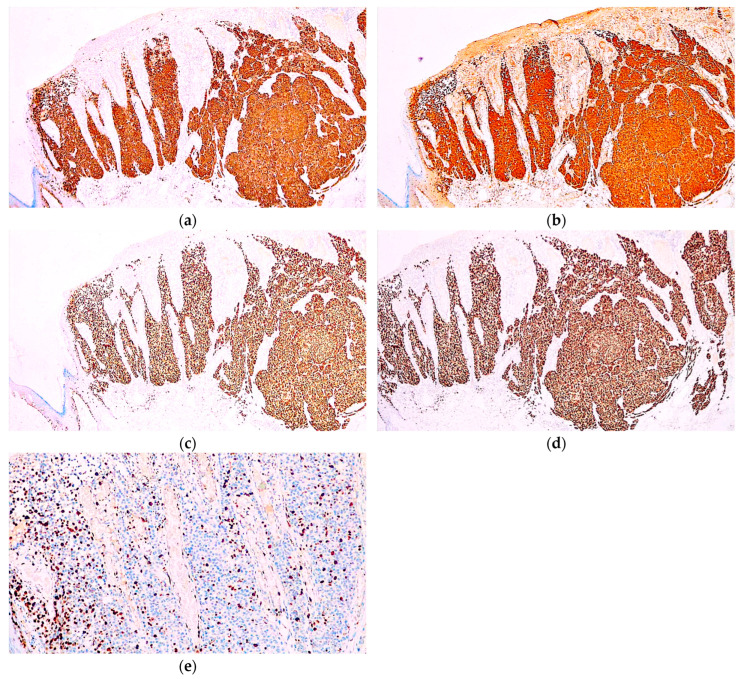
Immunohistochemical analysis of the cutaneous melanoma acknowledged diffuse positivity for (**a**) MelanA, (**b**) S100, (**c**) SOX10, and (**d**) PRAME. (**e**) The Ki67 immunoexpression was noted in 15% of the neoplastic cells.

The diagnosis was also confirmed immunohistochemically. The tumour cells were diffusely and intensely positive for multiple melanocytic markers: MelanA, S100, SOX10, and PRAME. Additionally, the Ki67 proliferation index was 15% (Figure 3).

**Figure 4:**
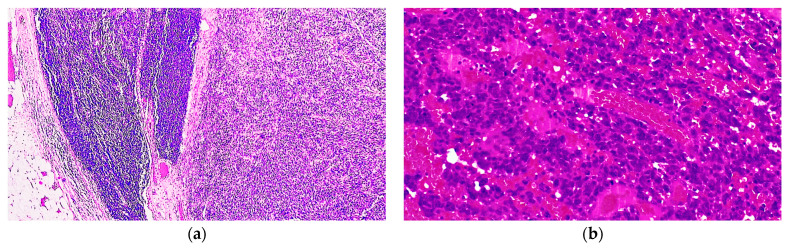
Histopathological analysis of the lymph node metastases revealed (**a**) the presence of a malignant spindle cell proliferation with a fascicular growth pattern (H&E, 40×) and (**b**) numerous ill-defined, branching vascular spaces (H&E, 400×).

Due to the depth of the tumour, a re-excision with 2 cm safety margins was performed. Later, the patient underwent a sentinel lymph node biopsy and contrast-enhanced computed tomography (CT) examination of the head and neck, and thoracic and abdominopelvic regions, but no regional or distant metastases were detected at that time. Therefore, no other treatment was initiated at this stage and the patient was called back for follow-up visits every 3 months. Unfortunately, at the 9-month visit in May 2022, suprascapular lymphadenopathy was detected. After surgical removal of the suprascapular mass, histopathological examination confirmed the presence of 11 lymph nodes, out of which 2 showed features of a sarcomatoid tumour proliferation with a fascicular growth pattern, encompassing ill-defined vascular spaces. The neoplastic cells were spindle-shaped, with no intracytoplasmic melanin and very frequent mitotic figures (Figure 4).

**Figure 5:**
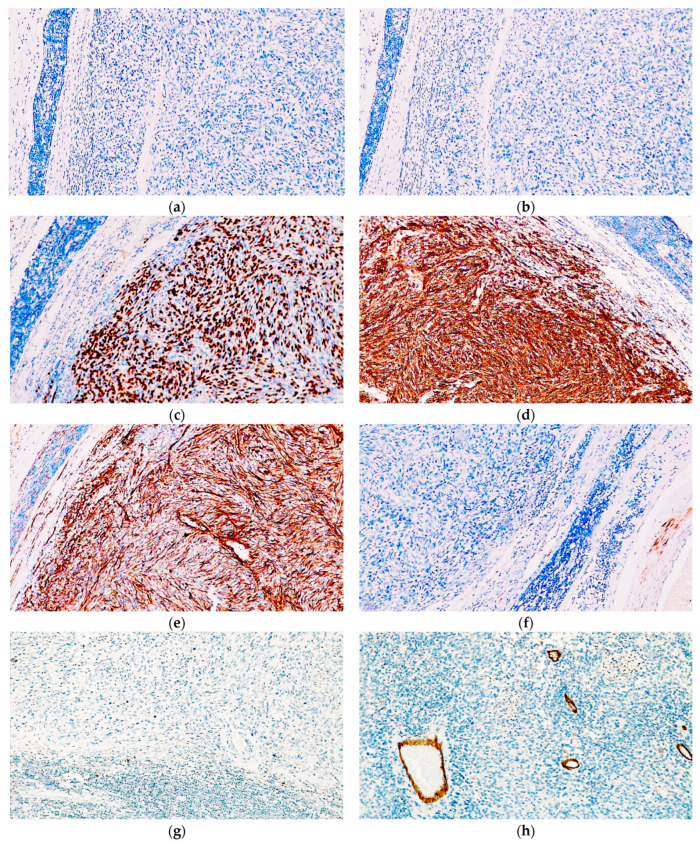

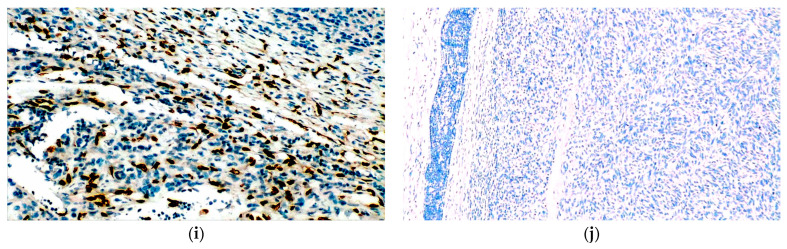
Immunohistochemical analysis of the lymph node revealed that the tumour cells were negative for (**a**) MelanA and (**b**) PRAME. However, the tumour cells displayed (**c**) strong positivity for SOX10 and showed strong and diffuse immunopositivity for (**d**) CD10 and (**e**) WT1, while (**f**) desmin, (**g**) CD99, and (**h**) CD31 were negative. (**i**) Positive ERG immunoreaction was noted in scattered tumour cells. (**j**) BRAF V600E was negative in the tumour cells.

Immunohistochemical analysis revealed that the tumour cells completely lacked expression of MelanA and PRAME, and focally expressed SOX10. On the other hand, CD10 was diffusely positive, and WT1 showed strong cytoplasmic expression. Desmin, CD99, and CD31 were negative, while ERG expression was noted in scattered tumour cells. Based on these findings, the diagnosis of metastatic melanoma with sarcomatoid features and areas of angiosarcomatous differentiation was established. Additionally, BRAF V600E immunohistochemistry was performed, but the test result was negative (Figure 5). 

Even though immunohistochemical analysis for *BRAF* mutations rendered negative results, genetic testing for *BRAF* mutations was also performed using an Idylla™ *BRAF* Mutation Assay cartridge (Biocartis, Mechelen, Belgium), revealing a wild-type phenotype. No other metastases were detected at the time. The patient was therefore started on treatment with pembrolizumab 200 mg every 3 weeks. 

**Figure 6:**
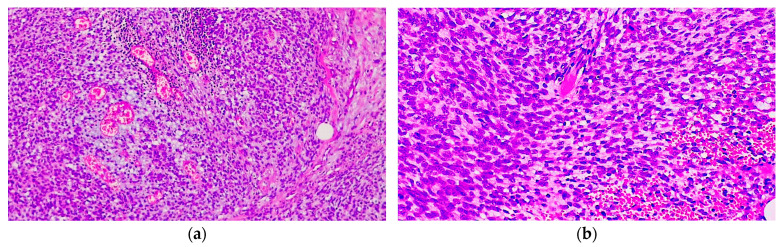
Histopathological examination of the skin lesion acknowledged (**a**) the presence of a solid sarcomatoid proliferation with extensive haemorrhage and (**b**) pleomorphic spindle cells surrounding anastomosing vascular spaces.

In April 2023, the patient presented again with a tumour recurrence at the site of the initial lesion. The tumour was once again removed and on histopathological examination this neoplasm also displayed sarcomatoid features, with solid sheets of highly pleomorphic spindle cells with amphophilic cytoplasm and numerous mitotic figures (22/HPF) and no intracytoplasmic pigment. Additionally, there were frequent blood lakes with haemorrhagic areas and ill-defined vascular channels (Figure 6).

**Figure 7:**
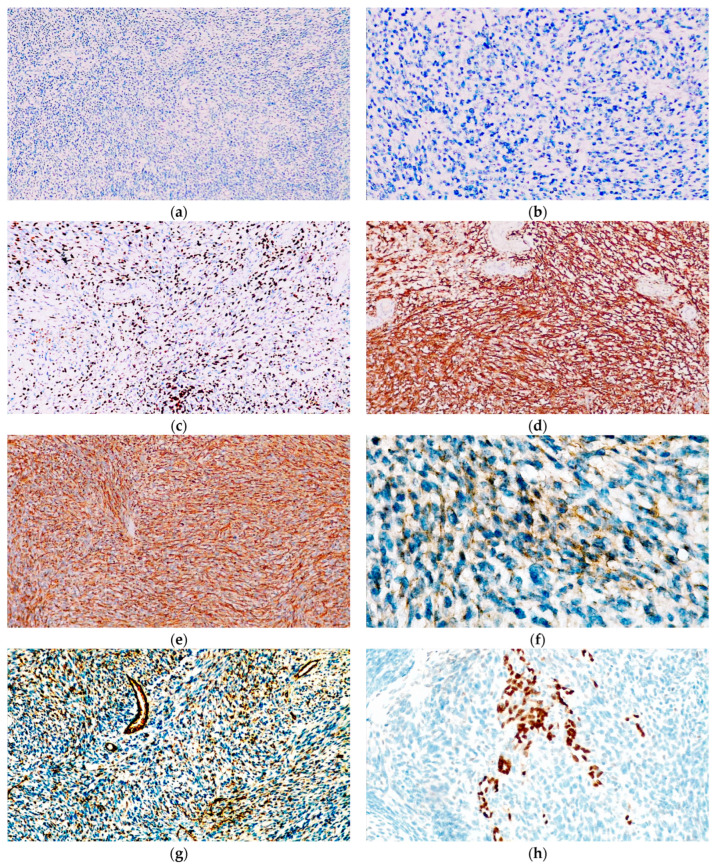
Immunohistochemical evaluation of the recurrent skin lesion proved that the tumour cells were not reactive for (**a**) MelanA and (**b**) PRAME. However, the tumour cells displayed moderate immunopositivity for (**c**) SOX10. Additionally, (**d**) CD10 and (**e**) WT1 were strongly and diffusely positive, while (**f**) CD99 was weakly positive in several areas. (**g**) CD31 was strongly positive in most of the neoplastic proliferation and (**h**) ERG was strongly positive in scattered tumour cells.

Immunohistochemical analysis revealed a similar profile to the lymph node metastasis. The tumour cells were completely negative for MelanA and PRAME while showing only focal SOX10 positivity. CD10 and WT1 were both diffusely positive, and CD99 was weakly positive in scattered tumour cells. Additionally, this time, the vascular marker CD31 was strongly and diffusely positive and ERG was strongly positive in scattered cells. These histopathological and immunohistochemical profiles are consistent with the diagnosis of recurrent sarcomatoid melanoma with genuine angiosarcomatous dedifferentiation (Figure 7). 

Due to tumour progression, treatment with pembrolizumab was deemed inefficient and the patient was switched to chemotherapy with dacarbazine but succumbed to widespread metastatic disease in February 2024. 

## 4. Discussion

Angiomatoid morphology in melanomas is exceptionally rare. To the best of our knowledge, there are only four cases reported in primary cutaneous melanomas, and three of metastatic melanomas. However, to this date, genuine angiosarcomatous dedifferentiation was noted in only one of the metastatic tumours [16,23,24,25,26,27,28]. The clinical, histopathological, and immunohistochemical features of these neoplasms are presented in Table 1.

By analysing the data presented in Table 1, it can be noted that the mean age of patients with angiomatoid melanomas is 65,375 years (SD = 13.04), and the male–female ratio is 3:1. These findings are highly concordant with our newly reported case, as the patient was a 65-year-old male.

As mentioned above, the case presented in this paper is only the second reported melanoma with angiosarcomatoid dedifferentiation highlighted by immunohistochemical expression of ERG and CD31, while lacking expression of MelanA and PRAME. In this context, the diagnosis of metastatic and recurrent melanoma was established due to the retained expression of SOX10. Similarly, Ambrogio F. et al. also concluded that SOX10 is the most reliable marker for diagnosing angiomatoid melanomas [24]. Furthermore, the metastatic tumour also expressed WT1, and Mehta A. et al. noted cytoplasmatic WT1 staining in a dedifferentiated metastatic melanoma, arguing that this pattern of expression may be useful for establishing the final diagnosis [29]. Therefore, in the right clinical context, ample immunohistochemical analysis for multiple melanocytic markers should be performed so as not to miss a diagnosis of dedifferentiated melanoma.

Concerning the expression of vascular markers, the other reported angiomatoid melanoma with positivity for ERG and CD31 completely lacked expression of S100, MelanA, and SOX10 and required molecular tests for confirmation [16]. As angiomatoid features are exceptionally rare in melanomas, little is known about the mechanisms behind this phenomenon. One of the possible explanations is that “mechanical stress” during the biopsy induces the formation of vascular spaces [24]. However, this explanation cannot be applied to our current case, in which differentiation was noted in both lymph node metastases and local recurrence and it was also confirmed by immunohistochemical expression of ERG and CD31. Therefore, angiosarcomatoid dedifferentiation in melanomas may be explained by a real phenotype shifting towards mesenchymal cells, which can be a means of cancer resistance [22,24]. We also favour this hypothesis due to the fact that the tumour presented in this study was highly aggressive, with poor response to systemic therapy. The disease was rapidly progressive and fatal.

In addition to immunohistochemical positivity for endothelial markers, the neoplastic cells of both the lymph node metastases and the recurrent skin tumour diffusely expressed CD10. Similar results have been reported by various authors in dedifferentiated melanomas [30,31,32,33] and CD10 has also been linked to promoting tumour progression and resistance to therapy [34]. Therefore, CD10 should be evaluated in metastatic melanomas, particularly in poorly differentiated lesions, as its expression could be a sign of phenotype shifting towards a more aggressive neoplasm.

Unlike CD10 diffuse expression, CD99 positivity was only observed in scattered cells in the recurrent skin tumour, demonstrating the transdifferentiation pathway followed by melanomas in their progression. In this respect, rare cases of dedifferentiated metastatic melanomas with CD99 have also been reported [35,36], highlighting the extraordinary heterogeneity of these neoplasms.

In our patient, PRAME analysis was performed for the first time in an angiomatoid melanoma, with the primary tumour expressing PRAME while the metastatic and recurrent lesions were negative for this marker. These results may seem surprising, since PRAME is regarded as one of the most reliable immunohistochemical markers for diagnosing dedifferentiated melanomas, either primary or metastatic [2,37,38]. However, the accuracy of these findings may be limited due to the low number of dedifferentiated melanomas with PRAME assessment. Further studies are required in order to fully define the utility of PRAME analysis in dedifferentiated melanomas, and in tumours with angiomatoid features in particular.

Dedifferentiated melanomas, especially in a metastatic context, may benefit from molecular analysis, not only for choosing the proper treatment but also for establishing the correct diagnosis [2,31,39,40]. In this respect, dedifferentiated melanomas usually retain melanoma-specific mutations even in metastatic settings, but such cases may also present epigenetic abnormalities characteristic of mesenchymal malignancies, thus matching the histopathological and immunohistochemical profile. Nevertheless, these modifications seem to be confined to the methylation signature, while specific copy number profiling appears to be retained in metastatic melanomas [41]. These observations are significant, as they highlight both the risk of misdiagnosing a metastatic melanoma based solely on methylation profile and the prospect of adapting treatment according to genetic abnormalities of the metastatic lesions. However, at present, the only genetic mutation that can benefit from target therapy is *BRAF* [2]. For this reason, our patient was tested for *BRAF* mutations, and following the negative results, no further molecular tests were performed.

Lastly, despite the valuable role of molecular analysis in dedifferentiated melanomas, such tests are expensive and still not readily available. Consequently, surrogate immunohistochemical markers for the most frequently encountered mutations, *BRAF* p.V600E and *NRAS* p.Q61, have been developed and are highly correlated with DNA analysis [42,43,44,45,46,47]. This correlation was also noted in the current case, with negative results in both immunohistochemical and molecular tests for *BRAF* mutations. 

Regarding the current state of treatment, the most commonly used therapies for locally recurrent or metastasised melanoma are immune checkpoint inhibitors and targeted therapies. Immune checkpoint inhibition is achieved by the use of anti-PD-1 agents (pembrolizumab, nivolumab) or by a combination treatment with the CTLA-4 inhibitor ipilimumab and the PD-1 inhibitor nivolumab. Targeted therapy uses BRAF inhibitors in combination with MEK inhibitors. This latter type of treatment is associated with a more rapid response but can only be used in melanomas that harbour an activating *BRAF* V600E mutation, and, unfortunately, resistance to treatment installs rapidly, after a median duration of 11 months [48,49,50,51,52,53]. 

The mechanisms that lead to targeted therapy resistance involve additional genetic mutations that activate the MAPK pathway, as well as non-genetic mechanisms, such as the remodelling of the extracellular matrix and transcriptional reprogramming. Remodelling of the extracellular matrix impedes T-cell migration and is implicated in resistance to immune checkpoint inhibitors as well. Additional alterations which lead to immune checkpoint inhibitor resistance are TGFβ-mediated downregulation of the expression of MHC class I molecules, decreased T-cell infiltration in the tumour, and loss of expression of melanoma differentiation antigens. In the future, this could have therapeutic implications. Molecules that inhibit the TGFβ pathway or collagen receptors could be added to therapeutic regimens in order to improve the response to targeted therapies and checkpoint inhibitors [10,11,12]. Nintedanib, a multikinase inhibitor and anti-fibriotic drug, shows promise in inhibiting extracellular matrix remodelling and preventing tumour relapse [54]. 

In the case of our patient, dedifferentiation occurred before the use of systemic treatments. Even though dedifferentiated melanomas have a grim prognosis, there have been some case reports of favourable responses to various treatment modalities, such as nivolumab [16] or interferon-α in combination with dacarbazine [28]. As of yet, there are no specific treatment recommendations for the treatment of dedifferentiated melanoma. Therefore, our patient was first treated with a PD-1 inhibitor, followed by conventional chemotherapy, but sadly did not respond. In the future, we can hope for more personalised therapies targeting factors that are implicated in the differentiation and cell survival of dedifferentiated melanoma. 

Advancing knowledge about the molecular mechanisms involved in melanoma tumourigenesis and in the development of resistance to treatment will hopefully lead to the development of new effective, more personalised treatment options for this type of cancer. New immune checkpoint inhibitors and targeted therapies are under development. Examples are the lymphocyte activation gene-3 (LAG-3) inhibitor relatlimab, RAF inhibitors (sorafenib, tovorafenib), CDK4/6 inhibitors (palbociclib), and inhibitors of the Met/HGF signalling pathway (crizotinib, tivantinib, quercetin) [55]. Talimogene laherperepvec is an already approved oncolytic viral therapy containing live herpes simplex virus 1 that can be used for the intralesional treatment of unresectable melanoma. It may also be useful as a neoadjuvant treatment [50,51,52,53,55]. Recently, lifileucel, an adoptive immune cell therapy with autologous ex vivo-expanded tumour-infiltrating lymphocytes, has been approved by the US Food and Drug Agency (FDA) for patients with advanced or unresectable melanoma progressing under other treatment modalities [56]. Other promising therapeutic modalities that are currently under development include chimeric antigen receptor T-cell (CAR T) therapy and cancer vaccines [55].

## 5. Conclusions

Divergent differentiation is a frequent yet poorly understood phenomenon in melanomas, posing real diagnostic and therapeutic challenges. Even though metastatic melanomas can exhibit various heterologous components, angiosarcomatous transdifferentiation is still extraordinarily rare. This case report documents the transition of a classic cutaneous melanoma to a highly aggressive sarcomatoid lesion as the disease progressed, highlighting the utility of ample histopathological, immunohistochemical, and molecular analysis, as well as discussing the prognostic meaning of phenotype shifting. 

## Figures and Tables

**Table 1:** Clinical, immunohistochemical, and molecular features of angiomatoid melanomas.

Author	Age	Gender	Tumour Location	Immunohistochemistry	Genetics
Ramos-Rodríguez G. et al. [22]	59	Male	Thigh	Positive: S100, HMB45, MiTF1, D2-40 Negative: CD31, p63, AE1/AE3 Ki-67: 5–10%	N/A
Ambrogio F. et al. [23]	87	Male	Cutaneous	Differentiated component: Positive: S100, MelanA, HMB45, SOX10 KI67: 5–6% Dedifferentiated component: Positive: SOX10 Negative: S100, MelanA, HMB45, CD31, CD34, and ERG KI67: 20%	*BRAF* V600E mutation
Fonda-Pascual P. et al. [24]	63	Female	Scapular region	Positive: S100, SOX9, HMB45 Negative: AE1/AE3, D2-40, CD31	*BRAF* V600E mutation
Baron J.A. et al. [25]	84	Male	Forehead	Positive: S100 Negative: HMB45	N/A
Adler M.J. et al. [26]	44	Male	Forehead metastases	Positive: S100, HMB 45, and vimentin	N/A
Zelger B.G. et al. [27]	56	Female	Subcutaneous metastases	Positive: S100, HMB45, MelanA, CD56	N/A
	61	Male	Axillary lymph node metastases	Positive: S100, CD56	N/A
Kilsdonk M.J. et al. [15]	69	Male	Inguinal lymph node metastases	Differentiated component: Positive: S100, MelanA, SOX10 Negative: ERG, CD31 Dedifferentiated component: Positive: ERG, CD31 Negative: S100, MelanA, SOX10	*NRAS* c.181_182delinsAG p mutation

N/A—not available.

## Data Availability

This article does not include any additional primary data besides the information already presented in the case report section.
